# Fish and Zooplankton Co‐Responses to Environmental Gradients Under Different Climate Change Scenarios

**DOI:** 10.1111/gcb.70845

**Published:** 2026-04-16

**Authors:** Cindy Paquette, Dario J. Di Girolamo, Jennifer Pham, Otso Ovaskainen, Stéphanie Gagné, Véronique Leclerc, Beatrix E. Beisner, Vincent Fugère, Zofia E. Taranu

**Affiliations:** ^1^ Département des Sciences de l’environnement et Centre de Recherche RIVE Université du Québec à Trois‐Rivières Trois‐Rivières Québec Canada; ^2^ Environmental Effects Research Division, Environment and Climate Change Canada Montreal Quebec Canada; ^3^ Department of Biological Sciences University of Quebec at Montreal Montreal Quebec Canada; ^4^ Interuniversity Research Group in Limnology (Groupe de Recherche Interuniversitaire en Limnologie; GRIL) Montréal Quebec Canada; ^5^ School of Biological Sciences Queen’s University Belfast Belfast Northern Ireland; ^6^ Department of Biology McGill University Montreal Quebec Canada; ^7^ Department of Biological and Environmental Science University of Jyväskylä Jyväskylä Finland; ^8^ Ministère de L’environnement, de la Lutte Contre Les Changements Climatiques, de la Faune et Des Parcs du Québec (MELCCFP) Montreal Quebec Canada

**Keywords:** biodiversity, fish, freshwater lakes, functional traits, hierarchical modeling of species communities, interaction networks, joint species distribution modeling, zooplankton

## Abstract

Climate change is reshaping freshwater ecosystems worldwide, yet the extent to which its effects differ across trophic levels remains poorly understood. Despite growing evidence of community restructuring, few studies have jointly examined how multiple trophic levels respond to climate forcing within a functional and network‐based framework. Herein, we assessed how fish and zooplankton communities respond to contemporary and projected end‐of‐century climate conditions using different shared socioeconomic pathways (SSP1‐2.6, SSP3‐7.0, and SSP5‐8.5). By means of joint species distribution models and ecological network analyses, we examined fish–zooplankton co‐responses to environmental gradients, quantified trait–environment relationships, and evaluated potential species interactions. We found that fish and zooplankton displayed distinct community and functional responses to current and projected climate gradients. Although both trophic levels were primarily influenced by climatic variables, fish exhibited stronger trait–climate relationships, including declining body size and increasing thermal tolerance with warming. Through ecological network analyses, we then demonstrated that freshwater communities tended to become more homogenized by 2100 under future climate change. Together, these results suggest contrasting climate sensitivities across trophic levels, potentially leading to trophic decoupling and functional reorganization of freshwater food webs at the regional scale. Our study highlights the value of combining trait‐based and network approaches to better anticipate community‐level responses to climate change.

## Graphical abstract

Climate change is reshaping freshwater ecosystems, but responses across trophic levels remain unclear. Here, we assessed fish and zooplankton responses to current and future climate scenarios in 156 lakes across Québec, Canada. Fish showed strong trait–climate relationships, with declining body size and increasing thermal tolerance, while zooplankton responses were weaker. Network analyses revealed increasing homogenization and reorganization of species associations by 2100. These patterns suggest trophic decoupling and functional shifts in freshwater food webs.

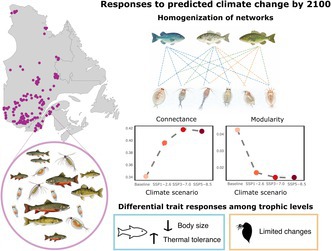

## 1 Introduction

Climate change is profoundly reshaping ecosystems, with rising temperatures and altered precipitation patterns impacting species distributions, interactions, and ecosystem functioning (Weiskopf et al. 2020). Biodiversity patterns often follow latitudinal gradients driven by productivity or energy availability, with higher species richness generally occurring at lower latitudes (Mannion et al. 2014). As such, predicting species range shifts according to species thermal tolerances has been well studied in various ecosystems (e.g., Sunday et al. 2012; Diamond 2018; Dahms and Killen 2023). However, anticipating how species' environmental niches will adjust to changing conditions, how functional traits mediate these responses, and how such changes may reorganize ecological networks remains a major challenge in global change ecology.

Species responses to climate change are particularly acute in lakes and rivers, where physical and biological processes are tightly coupled and climate‐driven stressors spread rapidly across trophic levels (Tickner et al. 2020). In aquatic ecosystems, the following three responses to climate warming are commonly observed: shifts in species' ranges toward higher altitudes and latitudes, changes in phenology, and an increased prevalence of small‐sized species (Daufresne et al. 2009; Ohlberger 2013; Comte and Grenouillet 2013; Woods et al. 2022). Such changes can decouple food webs and disrupt species interactions (Carroll et al. 2024). Among aquatic organisms, ectothermic groups like fish and zooplankton are particularly vulnerable to changes in temperature (Isaak and Rieman 2013), making them key indicators of climate impacts. As such, understanding how these groups respond to warming and the consequences for ecosystem structure and function is critical. Freshwater fish communities are critical for global food security, nutrition, and local economies, sustaining the livelihoods of millions of people (Lynch et al. 2016). Beyond their socioeconomic value, they also regulate food web dynamics and contribute to ecosystem resilience (Cooke et al. 2016). On the other hand, as secondary consumers, zooplankton provide an essential link in the food web by transferring energy from phytoplankton to fish (Jeppesen et al. 2011). However, fish and zooplankton have different ecologies (mobility, physiology, and thermal tolerance), which may result in contrasting responses to climate change.

Trait‐based approaches can help uncover these responses. Functional traits such as body size, thermal tolerance, and feeding strategy influence species fitness, community dynamics, and ultimately ecosystem functioning (Hébert et al. 2017). However, despite their value for predicting ecological responses to climate change, trait–climate interactions remain poorly understood in aquatic food webs. At the same time, climate‐driven habitat shifts can reshape ecological networks by altering species associations and interactions, leading to the gain or loss of links and, ultimately, the reorganization of community structure. For instance, recent work has shown that climate or land use change can reduce network connectance (i.e., the number of potential links among species) while simultaneously increasing modularity (i.e., the compartmentalization of species into subgroups) with implications for ecosystem stability (Merz et al. 2023; Bonnaffé et al. 2024; Dominique et al. 2025; Pham et al. 2025).

Few studies have considered vertical diversity spanning multiple trophic levels in aquatic ecology, yet they are necessary to explore shifts in food web stability and ecosystem functioning. Recent work has shown that environmental gradients can structure co‐responses across lake trophic levels, including among fish, zooplankton, and phytoplankton (Taranu et al. 2021; Di Girolamo et al. 2025); cladocerans, diatoms, and chironomids (Griffiths et al. 2021); and phytoplankton, zooplankton, and macroinvertebrates (Smith and Kirkwood 2025). Paquette, Gagné, Gaudet‐Boulay, et al. (2025) also demonstrated that zooplankton composition and functional traits were related to fish occurrences in lakes. Therefore, there is a need to disentangle these dynamics at the community level using integrative analytical approaches.

Joint species distribution modeling (JSDM) offers a promising framework to address this challenge, as it simultaneously accounts for abiotic filtering, biotic associations, spatial effects, and functional traits (Warton et al. 2015; Abrego et al. 2025). These models can also be used to forecast species correlations and trait responses under different environmental gradients, emerging as powerful frameworks in global change ecology for disentangling community responses to environmental change (Abrego et al. 2025). Joint species distribution models have previously revealed that water quality, lake morphometry, and interannual variability shaped plankton variation and niche dimensions in aquatic environments (Bolduc et al. 2026; Di Girolamo et al. 2025; Smith and Kirkwood 2025). However, surprisingly few studies have investigated the role of climate change in reshaping aquatic species' environmental niches, and the extent to which these responses are mediated by functional traits.

Here, we used fish and zooplankton occurrences from 156 lakes distributed across 17 degrees of latitude in Québec (Canada), one of the most lake‐dense regions in the world (Messager et al. 2016), to identify how climate change may reshape aquatic food webs. Specifically, we assessed whether fish and zooplankton diversity patterns are correlated, whether they respond to the same environmental gradients, and how network structure and trait–environment relationships may reorganize under baseline versus future climate scenarios based on different shared socioeconomic pathways (SSPs). We hypothesized that (i) zooplankton and fish diversity will be correlated, with higher *α*‐ but lower *β*‐diversity for both groups in southern Quebec as predicted by well‐documented latitudinal diversity gradients (Hillebrand 2004; Kraft et al. 2011). We further expected (ii) that current fish–zooplankton communities would exhibit strong co‐occurrence patterns because of trophic coupling or shared environmental niches (Taranu et al. 2021). Moreover, we predicted (iii) that species networks would become less connected and more compartmentalized under more extreme climate warming scenarios, as climate warming drives non‐random species losses and range shifts (Tylianakis and Morris 2017). Finally, since global warming has been demonstrated to benefit smaller freshwater organisms (Daufresne et al. 2009) and cause thermophilization of communities (Comte et al. 2021), we anticipated (iv) that functional traits of both fish and zooplankton would shift with climate change, notably through a replacement of large‐bodied, cold‐associated species with smaller‐bodied, thermophilic species along warming gradients.

## 2 Materials and Methods

### 2.1 Data Compilation

The Québec Ministère de l'Environnement, de la Lutte contre les Changements Climatiques, de la Faune et des Parcs (MELCCFP) sampled hundreds of lakes across the province of Québec since the late 1980s to ensure the conservation, enhancement, and protection of aquatic wildlife and its habitats (see Paquette, Gagné, Gaudet‐Boulay, et al. 2025; Paquette et al. 2026). Lakes in the MELCCFP monitoring program were selected to represent Québec's provincial fishing zones, resulting in greater sampling density in southern Québec and more limited coverage in northern regions with lower human density. This study uses a subset of 156 lakes from this database for which zooplankton data were available within the monitoring program, in addition to the fish survey data. These lakes were chosen to span the full north–south climatic gradient of the province and encompass a wide range of environmental conditions, providing broad geographic and ecological representation of Québec's freshwater systems.

In each lake, fish occurrence was compiled using different datasets from the MELCCFP, including fish species bycatch during standardized surveys (detailed in Service de la faune aquatique 2011), observations from non‐standardized surveys, and records from sport fishing. Because fishing efforts varied across datasets, data were harmonized using species‐level occurrences (presence–absence) rather than abundance, ensuring consistency across datasets. Fish taxa were then classified functionally using various traits related to trophic ecology and tolerance to climate change. Species were classified according to their trophic level (FishBase; https://www.shbase.se/), maximum body length (thereafter length), oral gape position (MoBd ratio from the FishMorph database; Su et al. 2021), and critical thermal maxima (CTmax; Comte and Olden 2017). Fish length and CTmax were used as continuous traits in our analyses, whereas trophic level and MoBd were categorized into discrete classes: primary consumers, secondary consumers, and top predators for trophic level; and bottom feeders, generalists or surface feeders for MoBd. Missing CTmax values for five species (*Hiodon tergisus, Notropis bifrenatus, Notropis stramineus, Prosopium cylindraceum*, and *Semotilus corporalis* ) were imputed using family means. While closely related species were found to exhibit similar fish thermal tolerance (Comte and Olden 2017), we acknowledge that this imputation may mask species‐specific deviations from family averages.

To enhance spatial overlap between zooplankton and fish sampling sites, we integrated data from three separate pelagic crustacean zooplankton datasets, including the MELCCFP fish monitoring program (*n* = 120), the MELCCFP Biodiversity Monitoring Network (BdQc; *n* = 23), and the NSERC Canadian Lake Pulse Network project (Huot et al. 2019, *n* = 13). In all three programs, zooplankton were sampled at the lake's deepest point during the summer stratification with a 53–100 μm Wisconsin net, towed vertically from the bottom to the surface. Samples were then narcotized and fixed in 70% ethanol. For consistency, the sample count criteria of 200 zooplankton individuals of the same taxa or 1000 individuals total for each lake was met in all three datasets. Abundance data was converted in presence–absence format to ensure consistency with the fish datasets. Preliminary analyses indicated no significant differences in zooplankton richness among datasets (results not shown). Functional trait attribution followed the original Barnett et al. (2007) and updated Paquette et al. (2021) classification including habitat (littoral, pelagic, or intermediate), resource acquisition [B (Bosmina)‐filtration, C (Chydorus)‐filtration, D (Daphnia)‐filtration, S (Sida)‐filtration, stationary suspension, current cruiser or raptorial], trophic group (carnivore, herbivore, or omnivore), and size (measured as body length of up to 10 individuals per species per sample). Although size was used as a continuous variable in our models, for visualization, species were assigned a size class of small or large using a cutoff of 0.5 mm (Paquette et al. 2021).

Water quality variables, including oxygen (surface, hypolimnion, and water column mean), temperature (surface, hypolimnion, and water column mean), pH, conductivity, and thermocline depth, were measured in situ using a multiparameter probe (Yellow Spring Instruments, Ohio), while water transparency was estimated using a Secchi disk. All variables were collected during MELCCFP fish surveys at the deepest point of each lake. Water quality parameters were averaged over time for each lake when multiple samplings occurred. The LakeAtlas database (v.1.0; Lehner et al. 2022) was used to extract lake morphometric variables for each lake, including lake area, circularity, mean depth, volume, discharge, water residence time, shoreline length, shoreline development, elevation, watershed slope, and watershed area. The watershed estimated human population density as of 2018 was also obtained for each lake using LakeAtlas.

Climate data were extracted for each sampling location under baseline and future climate conditions considering three different shared socioeconomic pathways (SSP1‐2.6, SSP3‐7.0, and SSP5‐8.5) using the WorldClim version 2.1 and the Coupled Model Intercomparison Project Phase 6 (CMIP6) framework (https://www.worldclim.org; O'Neill et al. 2016; Meinshausen et al. 2020). Briefly, SSP1‐2.6 represents a sustainability‐focused scenario with limited warming < 2°C by 2100. SSP3‐7.0 describes a regional rivalry scenario leading to moderately high emissions, while SSP5‐8.5 reflects a fossil‐fueled development pathway and very high emissions. Baseline conditions represented averaged data from 1970 to 2000. Future climate conditions were extracted for the 2081–2100 period and averaged over six different global climate models: MIROC6, MPI, IPSL, MRI, ACCESS, and UKE. Climate variables selected were minimum air temperature, maximum air temperature, annual air temperature, precipitation seasonality and annual precipitation, using 5 min resolution.

To stabilize variance in the predictor distributions, environmental variables were first transformed using either log or square‐root transformations. We then screened the dataset a priori for highly correlated explanatory variables (Pearson *r* > 0.9), removing shoreline development, lake volume, shore length, discharge, and annual mean temperature. Finally, the few missing values were imputed using the “mice” R package (van Buuren et al. 2023). All statistical analyses were performed in R v. 4.1.0 (R Core Team 2021).

#### 2.1.1 Diversity Within and Among Trophic Groups

To compare patterns in fish and zooplankton diversity across our lakes, we used bivariate maps with taxonomic richness and local contribution to *β*‐diversity (LCBD), a measure of each lake's uniqueness in community composition. Local contribution to *β*‐diversity values were computed separately for fish and zooplankton using species presence–absence data and the Jaccard index with “adespatial” R package (Legendre and De Cáceres 2013; Legendre 2014). Holm's procedure (Borcard et al. 2018) was used to assess significant LCBD sites after correcting for multiple testing. To examine the relationship between LCBD values and species richness for fish and zooplankton, and to evaluate whether sites were more unique due to extreme highs or lows in species richness (Legendre and Legendre 2012), we regressed LCBD against richness within each trophic group. To then compare richness and turnover across trophic groups, we regressed fish richness versus zooplankton richness, and fish LCBD versus zooplankton LCBD. For all regressions, linear and quadratic models were tested, and the models with the highest *R* ^2^ were retained.

#### 2.1.2 Species Co‐Responses to Environmental Factors

A random forest (RF) model was used to reduce multicollinearity and identify predictors of interest for fish and zooplankton occurrence. We implemented a multivariate RF using the “randomForestSRC” R package (Ishwaran and Kogalur 2025), where the response matrices consisted of combined fish and zooplankton presence‐absence values (excluding rare species, i.e., species occurring in only one lake; *n* = 67 and 53 species for fish and zooplankton, respectively after exclusion), and the predictors included all environmental variables. Variable importance for each species was weighted using misclassification error rates and then averaged across all species to obtain a global variable importance. The top 10 predictors were then retained for subsequent Hierarchical Modeling of Species Community (HMSC; Ovaskainen et al. 2017; Ovaskainen and Abrego 2020), a joint species distribution modeling framework. This threshold was selected to optimize the number of variables relative to the sample size (~15 observations per variable). We also computed the multivariate RF separately on zooplankton and fish species, obtaining the exact same set of top predictors (results not shown) and thus present the single model for simplicity.

HMSC was applied to model the combined fish and zooplankton species co‐occurrences while accounting for environmental predictors, species traits, and spatial autocorrelation. We used the “Hmsc” R package (Tikhonov et al. 2020) for model fitting, with a binomial distribution for species presence‐absence data, excluding rare species. The retained predictors from the multivariate RF formed the fixed effects in the model and random effects included sampling sites and spatial coordinates to account for hierarchical sampling structure and latent spatial patterns. Although longitude and latitude were selected among the top 10 most important variables, they were not added as fixed terms since they were included as random levels in the model, resulting in a total of eight fixed terms in the HMSC (maximum and minimum air temperature, watershed area, lake area, population density, annual precipitation, precipitation seasonality, and elevation). Fish trophic level and MoBd, as well as zooplankton trophic group, habitat and feeding type were included in the model as factor variables. Fish length, fish CTmax, and zooplankton length were treated as numerical variables and were log‐transformed and standardized prior to analysis. The model was run with two Markov Chain Monte Carlo (MCMC) chains, each generating 1000 posterior samples after a transient phase of length transient = 500 × thin and a thinning interval of 5 (thin = 5, samples = 1000, nChains = 2, transient = 2500, verbose = 2500). Model fit was evaluated using the MCMC convergence, while explanatory power was estimated using the model Tjur's *R* ^2^ (pseudo *R* ^2^ value for binary HMSC models).

We used variation partitioning to quantify the relative importance of fixed environmental predictor and latent spatial variables to fish and zooplankton occurrence patterns. For each species, the proportion of explained variance was adjusted by each species' Tjur's *R* ^2^ to better reflect the absolute explanatory power of each predictor, following the method from Di Girolamo et al. (2025). Species responses to environmental gradients (*β* coefficients) and the effects of functional traits on species niches (γ coefficients) were visualized using heatmaps, both using posterior probabilities thresholds > 90%. Fish‐zooplankton pairwise co‐occurrences were examined as model residual variation (ω coefficients) with posterior probabilities > 95% after accounting for the effect of environmental variables. Chord diagrams were used to visualize statistically supported residual associations between fish and zooplankton species using the “circlize” R package (Gu et al. 2014).

#### 2.1.3 Networks and Trait Changes Under Different Climate Scenarios

For both baseline and future climate scenarios, species occurrence probabilities were estimated from posterior predictive distributions using prepareGradient() and predict.Hmsc() R functions, both from “Hmsc” package. The site‐by‐species predictive probability matrices were then used to construct networks by computing Spearman's species‐pairs correlation coefficient using “Hmisc” package (Harrell 2023). Final networks for each scenarios included only species correlations with *p*‐values < 0.01. Each scenario network (baseline, SSP1‐2.6, SSP3‐7.0, and SSP5‐8.5) was described using key network metrics: number of taxa (i.e., nodes), Spearman's correlation coefficient between each taxa pair (i.e., edges), network connectance (i.e., edge density), average path length for all node pairs (i.e., mean distance), number of communities (i.e., edge betweenness community) and modularity. All network metrics were computed with “igraph” R package (Csárdi et al. 2024), following the method from Pham et al. (2025). Differences in parameters among networks were visualized using faceted line plots showing scenario‐specific trends in each metric. Changes in species co‐occurrences between each climate change scenario and baseline networks, including the number of associations lost, gained or retained, were visualized using a barplot.

To facilitate the interpretation of trait–environment relationships at the community scale, we computed community‐weighted means (CWMs) of functional traits for each site using predicted occurrence probabilities for each scenario. CWMs were calculated by weighting species' trait values by their relative probability of occurrence within each community, providing a summary measure of expected functional trait composition at the assemblage level (Shipley et al. 2006; Burner et al. 2023). We then visualized CWMs in relation to baseline and future climate gradients using scatterplots and smoothed regression lines. Finally, fish species richness was plotted against baseline maximum air temperature and across fish length gradients to disentangle fish length CWM trends.

## 3 Results

### 3.1 Diversity Patterns

**FIGURE 1:**
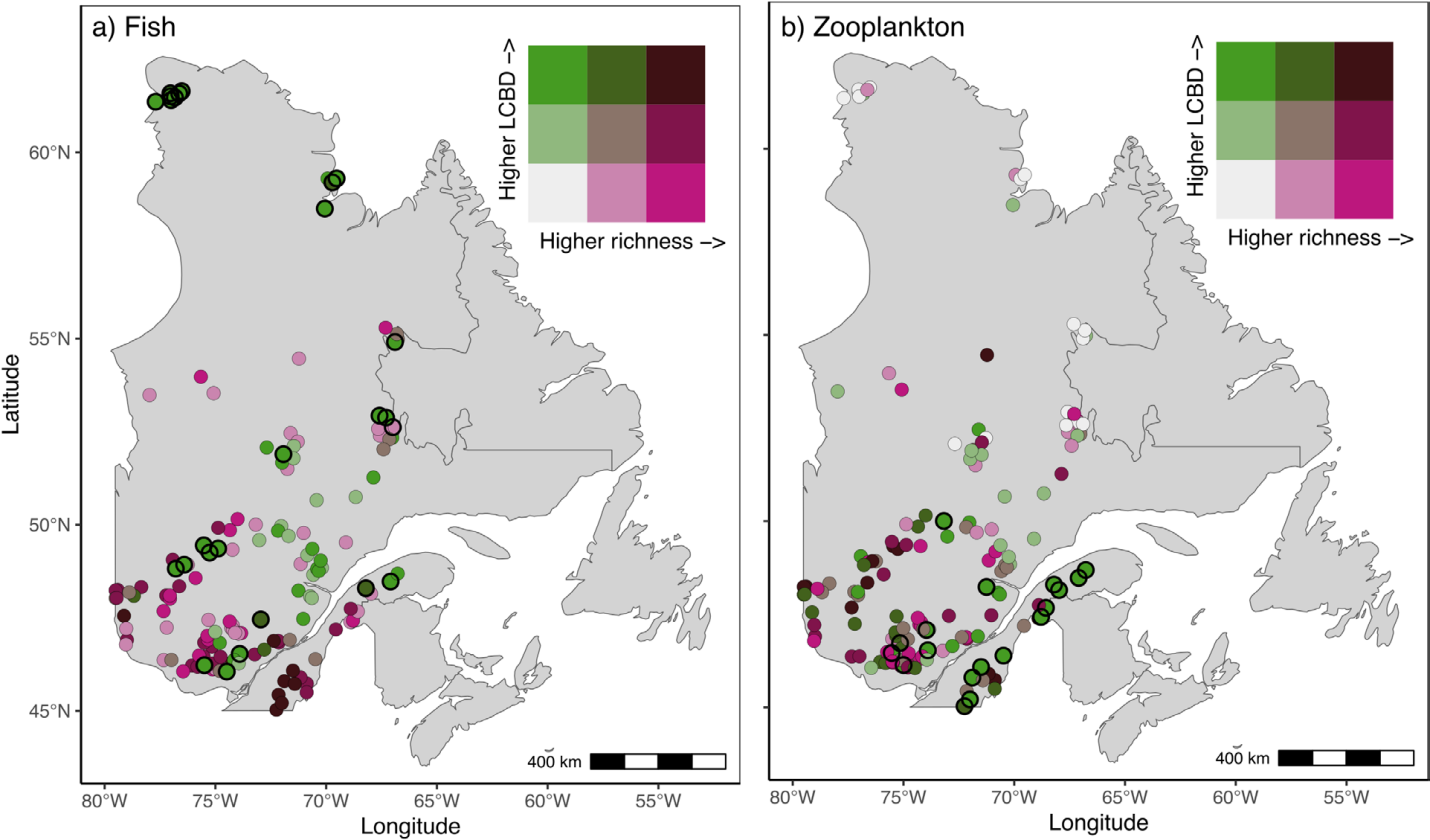
Bivariate map showing fish (a) and zooplankton (b) species richness and the local contribution to *β*‐diversity LCBD with the location of the 156 lakes sampled lakes. Sites with significant LCBD values are shown with black borders. Map created using Mercator projection (WGS84 CRS). Map lines delineate study areas and do not necessarily depict accepted national boundaries.

**FIGURE 2:**
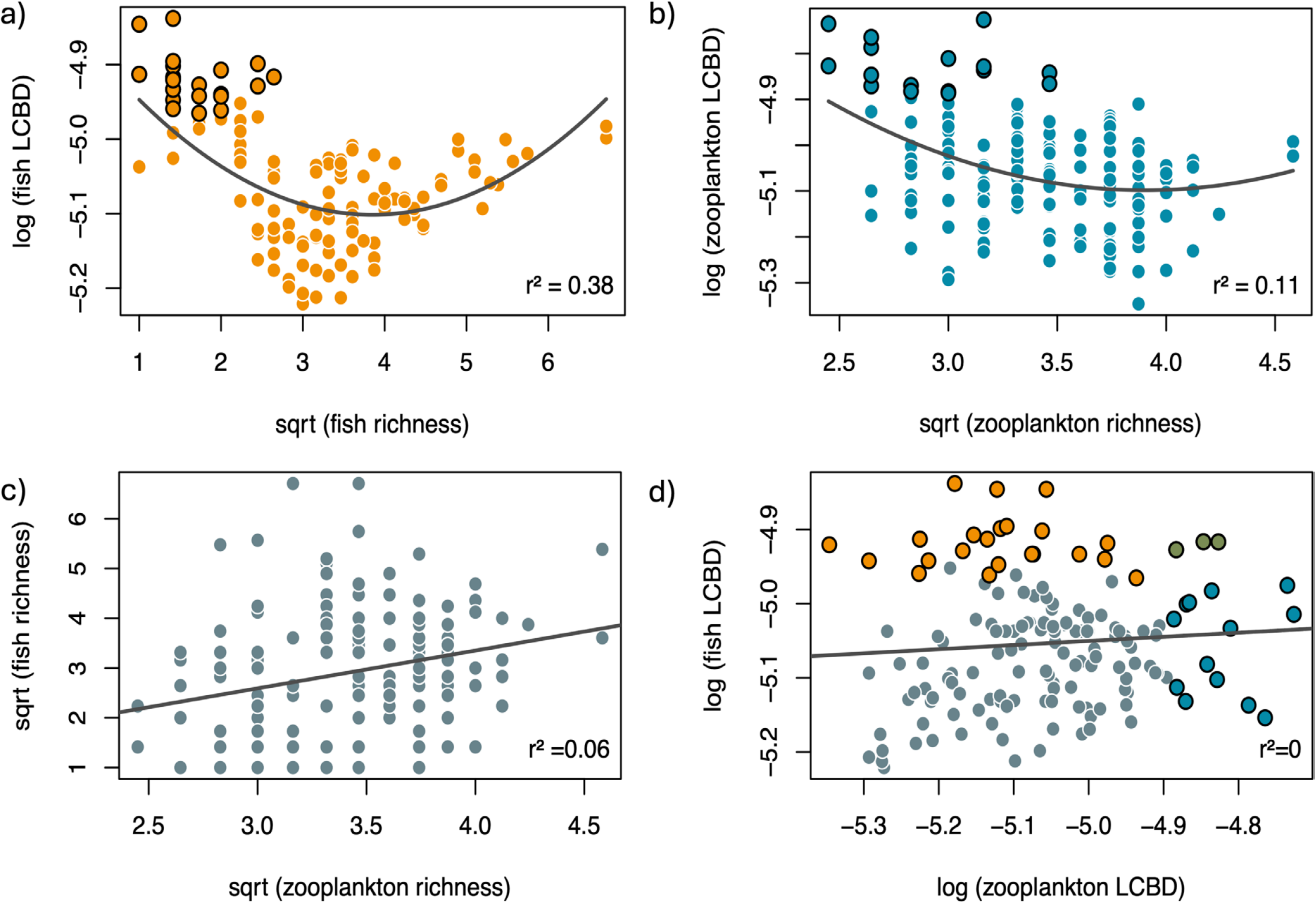
Relationships between local contribution to *β*‐diversity (LCBD) and species richness for fish (a) and zooplankton (b), and between fish‐zooplankton richness (c) and LCBD (d). In panels (a), (b), and (d), sites with significant LCBD values are shown with black borders and respective colours.

Fish communities were composed of 97 species, with local richness ranging from 1 to 45 species. The richness gradient followed a latitudinal pattern, with maximal diversity in southern Québec (Figure 1a). Conversely, fish LCBD was maximal in northern Québec, although significant LCBD sites were scattered across the province (Figure 1a), resulting in an overall quadratic richness‐LCBD relationship (*R* ^2^ = 0.38; Figure 2a). Sites with both high *α*‐ and *β*‐diversity values (including significant LCBD sites) were mostly located in southern Québec (Figure 1a). On the other hand, zooplankton richness ranged from 6 to 21 species, within a total of 70 identified species. Zooplankton diversity had different patterns than the fish communities, with zooplankton *α*‐ and *β*‐diversity both showing maximal values in southern regions of the province (Figure 1b). A quadratic model also provided the best fit to model zooplankton richness‐LCBD patterns, although the model strength was smaller than for fish (*R* ^2^ = 0.11; Figure 2b). When comparing richness and LCBD values between fish and zooplankton communities, we noted no clear relationship (*R* ^2^ = 0.06 and 0.00 respectively; Figure 2c,d). Only three sites located in southern Québec had significant LCBD values for both fish and zooplankton communities.

### 3.2 Species Responses to Environmental Gradients

RF models were used to identify and select predictors of interest across all fish and zooplankton species after removing rare species. In order of declining importance, the top 10 variables were maximum air temperature, latitude, minimum air temperature, longitude, watershed area, lake area, population density, annual precipitation, precipitation seasonality, and elevation (Figure S1). HMSC was then used on the top RF variables (excluding lake coordinates, which were included as random factors) to assess fish‐zooplankton species co‐responses to environmental gradients, while accounting for spatial factors and species traits.

The HMSC analysis showed that fish and zooplankton occurrences were associated with the environmental variables selected by the random forest model with 27% of explained variation. Species‐specific responses to environmental covariates, as captured by the *β* parameters, revealed varying ecological niches among taxa (Figure S2). Maximum temperature and watershed area had overall positive effects on fish and zooplankton species occurrences. Fish species were found to generally respond positively to minimum air temperature, while only a few zooplankton taxa responded, most of them negatively. Lake area showed a positive trend with fish species, while the relationship varied across zooplankton species. The responses of fish and zooplankton varied among species for population density, annual precipitation and precipitation seasonality. Finally, lake elevation showed positive relationships with zooplankton species, while relationship directionality varied for fish.

**FIGURE 3:**
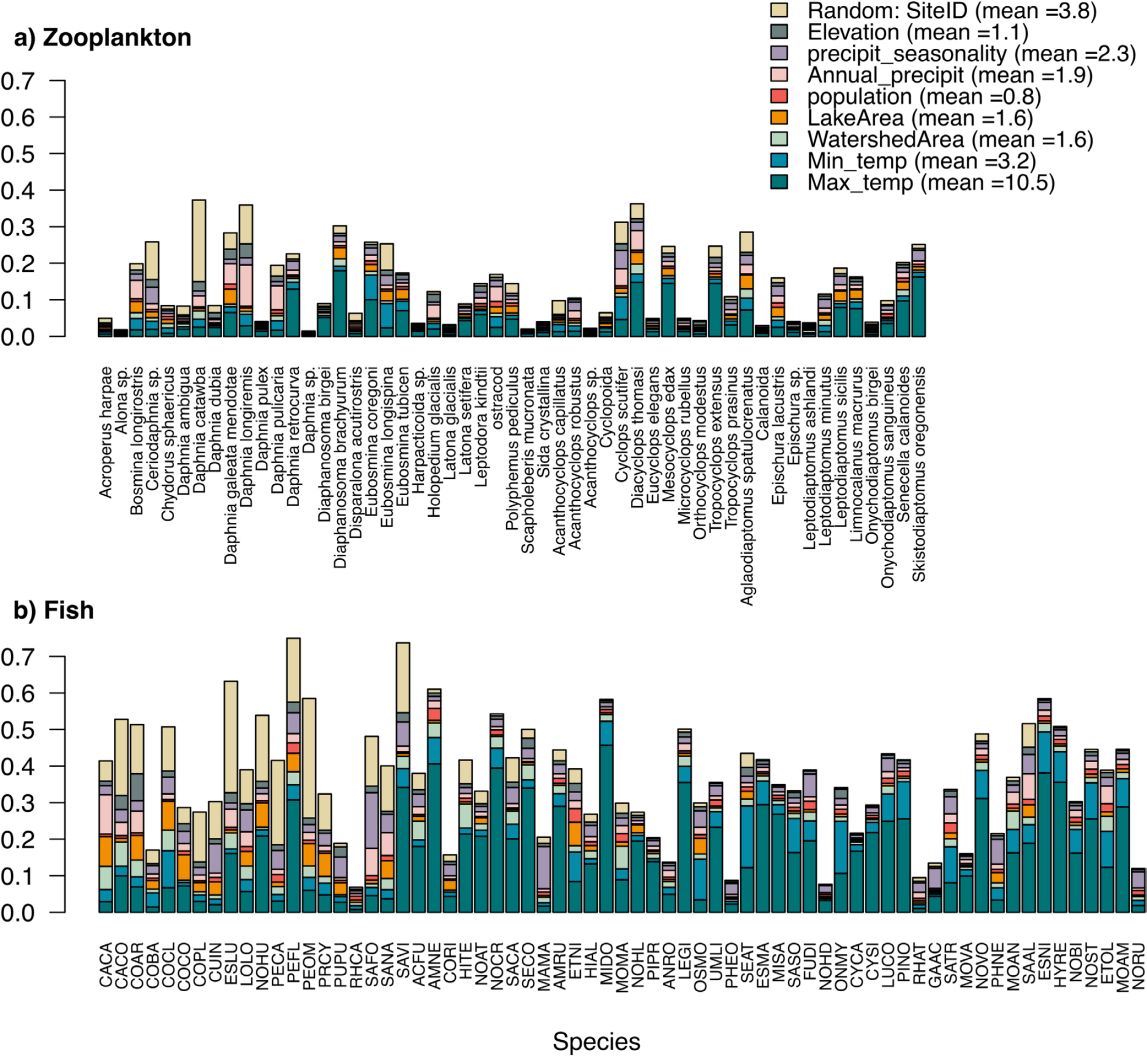
Variation partitioning of zooplankton (a) and fish (b) occurrences based on environmental predictors included in the HMSC, adjusted by species‐specific *R* ^2^ values. Full fish species names associated with the taxonomic codes are described in Table S1.

Variance partitioning adjusted by species Tjur's *R* ^2^ revealed that overall fish species had a larger proportion of variance explained by the selected environmental variables than did zooplankton species (Figure 3). Across all taxa, climate variables consistently emerged as the most important predictors (10.5%, 3.2%, 1.9%, and 2.3% of mean variance explained by maximum air temperature, minimum air temperature, annual precipitation and precipitation seasonality, respectively), while lake area, watershed area, lake elevation, and human population density had relatively minor effects for most species (1.6%, 1.6%, 1.1%, and 0.8% of mean variance explained, respectively). The mean variation attributable to site coordinates accounted for the second largest proportion of variance in species occurrence, with 3.8%. Spatial random effects were mainly captured by the first two latent spatial factors, with median spatial scales (*α*) of 101.30° (95% CI: 60.78–162.07) and 60.78° (95% CI: 0–222.85), respectively. Factors 3 and 4 had no support for spatial structure, with median estimate of zero and wide credible intervals, indicating high uncertainty.

### 3.3 Trait Influence on Species Niche

Distinct associations between species traits and environmental covariates were revealed by *γ* parameters (Figure S3). Overall, fish traits exhibited stronger associations with environmental variables than zooplankton traits. Maximum temperature was significantly associated with all fish traits, showing positive effects on fish length, CTmax, surface feeders, and generalists (species with no preference for surface vs. bottom feeding). On the other hand, negative effects of maximum temperature were observed on top predators and secondary consumers. Minimum temperature was positively related only to fish CTmax. Watershed area was positively associated with fish length and CTmax, while lake area and population size influenced CTmax negatively and positively, respectively. Precipitation variables also shaped fish traits, with total annual precipitation negatively affecting surface feeders and precipitation seasonality positively related to fish length. Both fish CTmax and length were positively associated with elevation. In contrast, few zooplankton traits showed clear associations. Carnivores were negatively linked to population density; littoral species were negatively associated with population density but positively with annual precipitation; and intermediate‐habitat species were positively related to watershed area and negatively to lake area.

### 3.4 Fish‐Zooplankton Residual Associations

**FIGURE 4:**
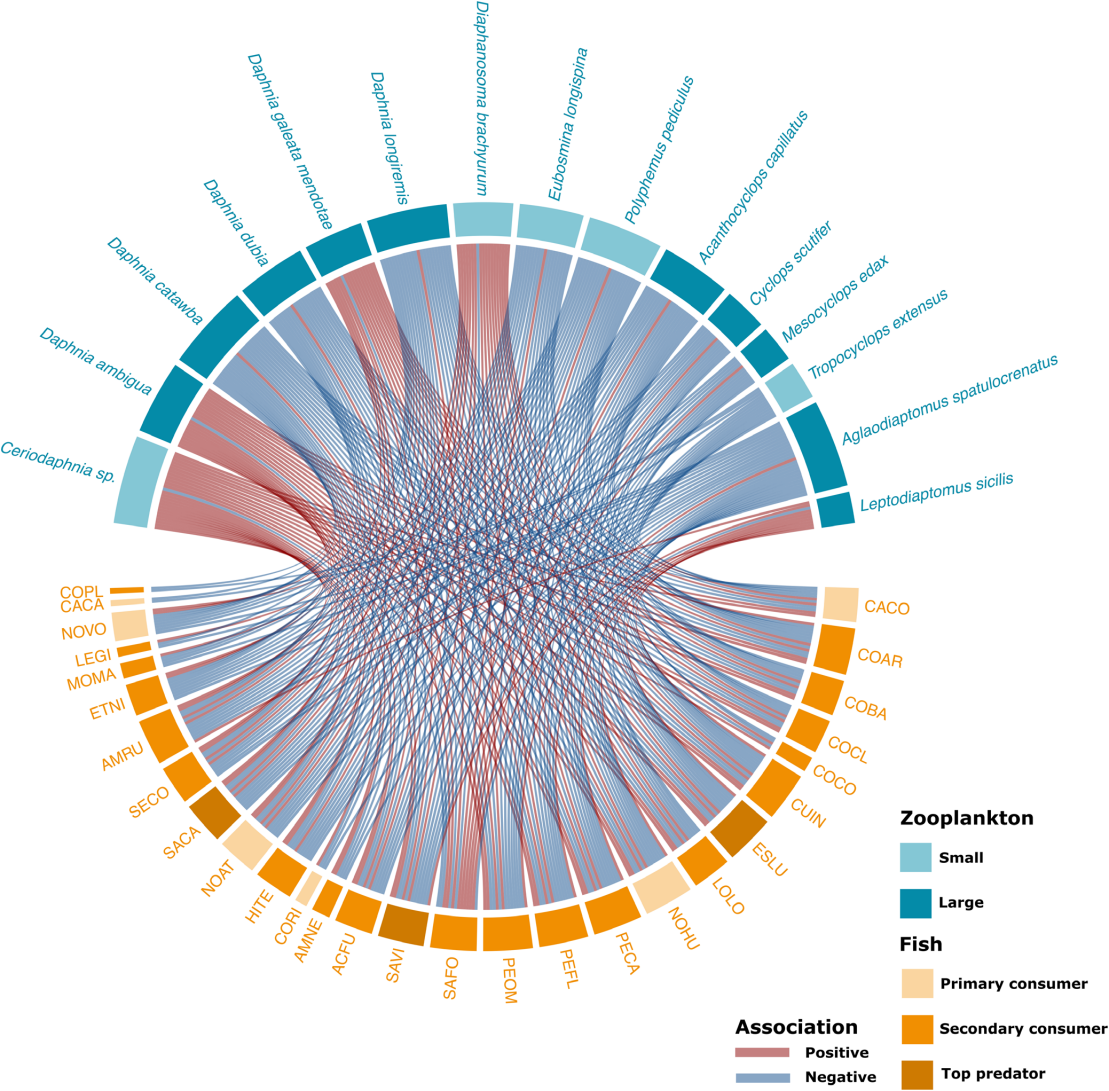
Chord Diagram showing positive (red) and negative (blue) HMSC significant residual associations between fish (orange) and zooplankton (blue). Full fish species names associated with the taxonomic codes are described in Table S1.

Statistically supported residual associations were most frequent between fish and zooplankton species and were mostly positive (6.2% positive vs. 4.0% negative; Figure 4). In contrast, fish–fish (0.9% positive, 0.1% negative) and zooplankton‐zooplankton (0.7% positive, 0.6% negative) residual associations were rare. While 28 different fish species had statistically supported associations with zooplankton species, only 15 zooplankton species had statistically supported residual associations with fish species. Most zooplankton species showed negative residual associations with fish species, although *Ceriodaphnia* sp., *Daphnia ambigua, Daphnia galeata mendotae*, *Diaphanosoma brachyrum* and *Leptodiaptomus sicilis* showed mainly positive associations (Figure 4; Figure S4). Brook trout (SAFO) was the only fish species to show more positive than negative associations with zooplankton species, with the direction of these associations always being opposite to that observed for all other fish species (Figure 4; Figure S4).

### 3.5 Networks, Trait and Species Changes Under Different Climate Scenarios

**FIGURE 5:**
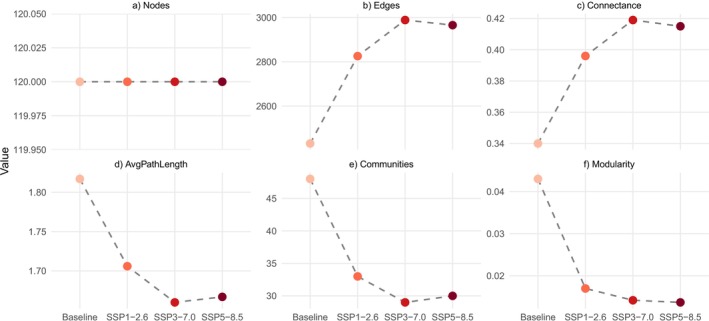
Scenario‐specific ecological network trends in number of nodes (a), number of edges (b), connectance (c), average path length (d), number of communities (e), and modularity (f), across baseline and climate scenarios SSP1‐2.6, SSP3‐7.0, and SSP5‐8.5.

Network parameters varied across the baseline and climate change scenarios (Figure 5). The number of significant nodes remained stable at 120 across all scenarios. A consistent increase in the number of edges and in connectance was observed from the baseline to SSP3‐7.0, followed by a slight decrease under SSP5‐8.5. Changes in the number of edges (i.e., significant species co‐responses) were primarily driven by species gains, with comparatively fewer species losses relative to the baseline scenario (Figure S5). In contrast, average path length, number of communities and modularity decreased until SSP3‐7.0, then stabilized or increased slightly under SSP5‐8.5.

**FIGURE 6:**
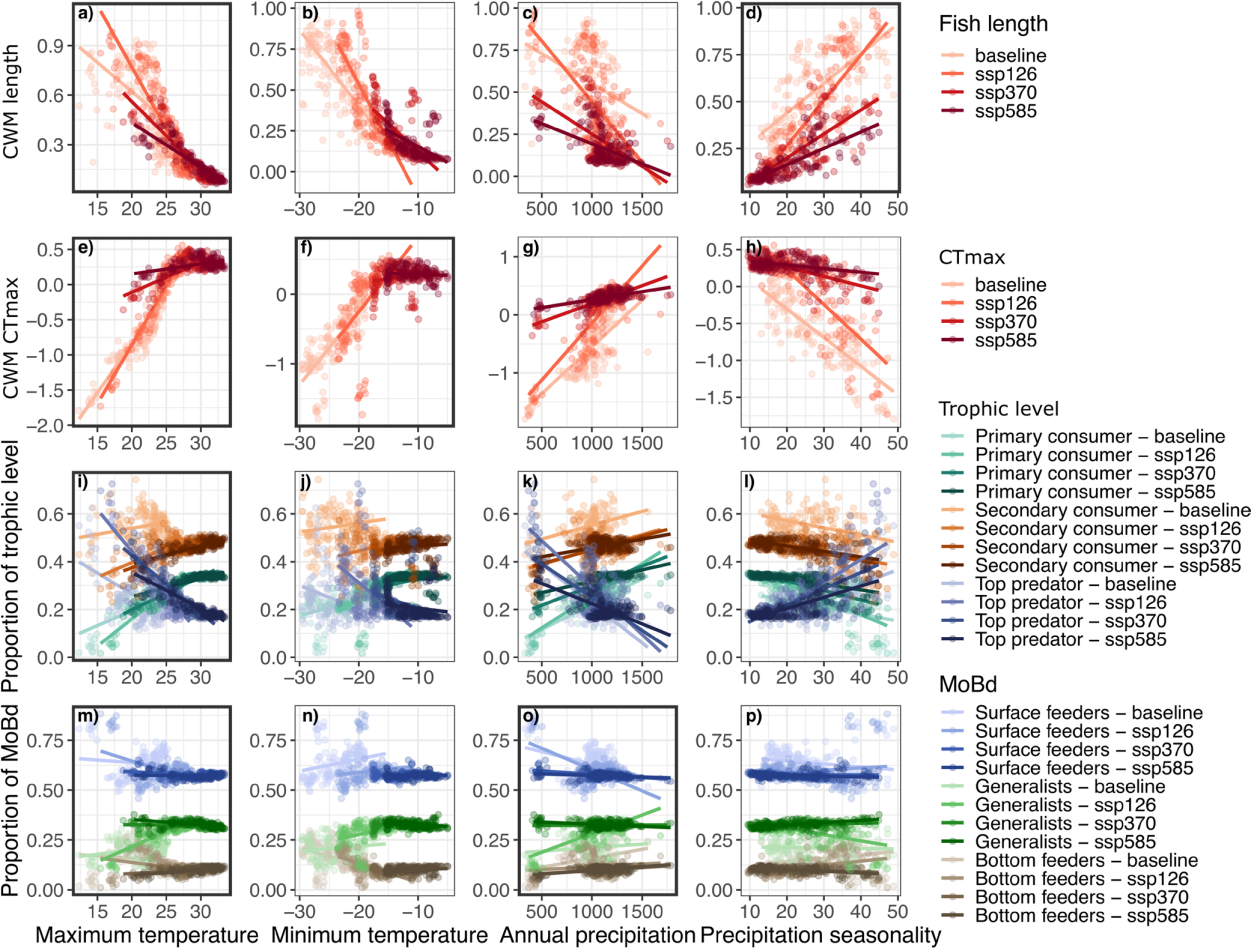
Responses of fish traits to climate covariates based on the proportion of trait types (categorical traits) and community‐weighted means (CWM; quantitative traits) under baseline and future climate scenarios (SSP1–2.6, SSP3–7.0, and SSP5–8.5). Darker shades represent more extreme climate scenarios, while lighter shades represent baseline conditions. Panels with thicker outlines (a, d, e, f, i, m, o) indicate significant trait–environment relationships in baseline conditions (γ plot).

Comparing across baseline and climate change scenarios, climate‐trait interactions were detected based on species CWMs. Fish body lengths decreased, while CTmax increased in response to rising temperatures and altered precipitation patterns (Figure 6). For fish trophic level, rising temperatures and altered precipitation patterns decreased the proportion of secondary consumers but increased the proportion of primary consumers and had little effect on top predators. Moreover, the proportion of surface feeders fish species decreased slightly with climate change, and the proportion of generalists increased, while bottom feeders remained generally unchanged. For all fish functional traits, CWM–climate relationships weakened under more extreme scenarios, as indicated by flatter slopes and narrower temperature gradients.

Since the *γ* plot and CWM showed opposite responses of fish length to temperature (positive γ relationship, but negative CWM slopes), we examined fish richness along the baseline temperature gradient. This revealed that while small fish species have weaker responses to temperature than large fish (positive *γ*), overall species richness increased with temperature, reducing the community mean body length (Figure S6).

**FIGURE 7:**
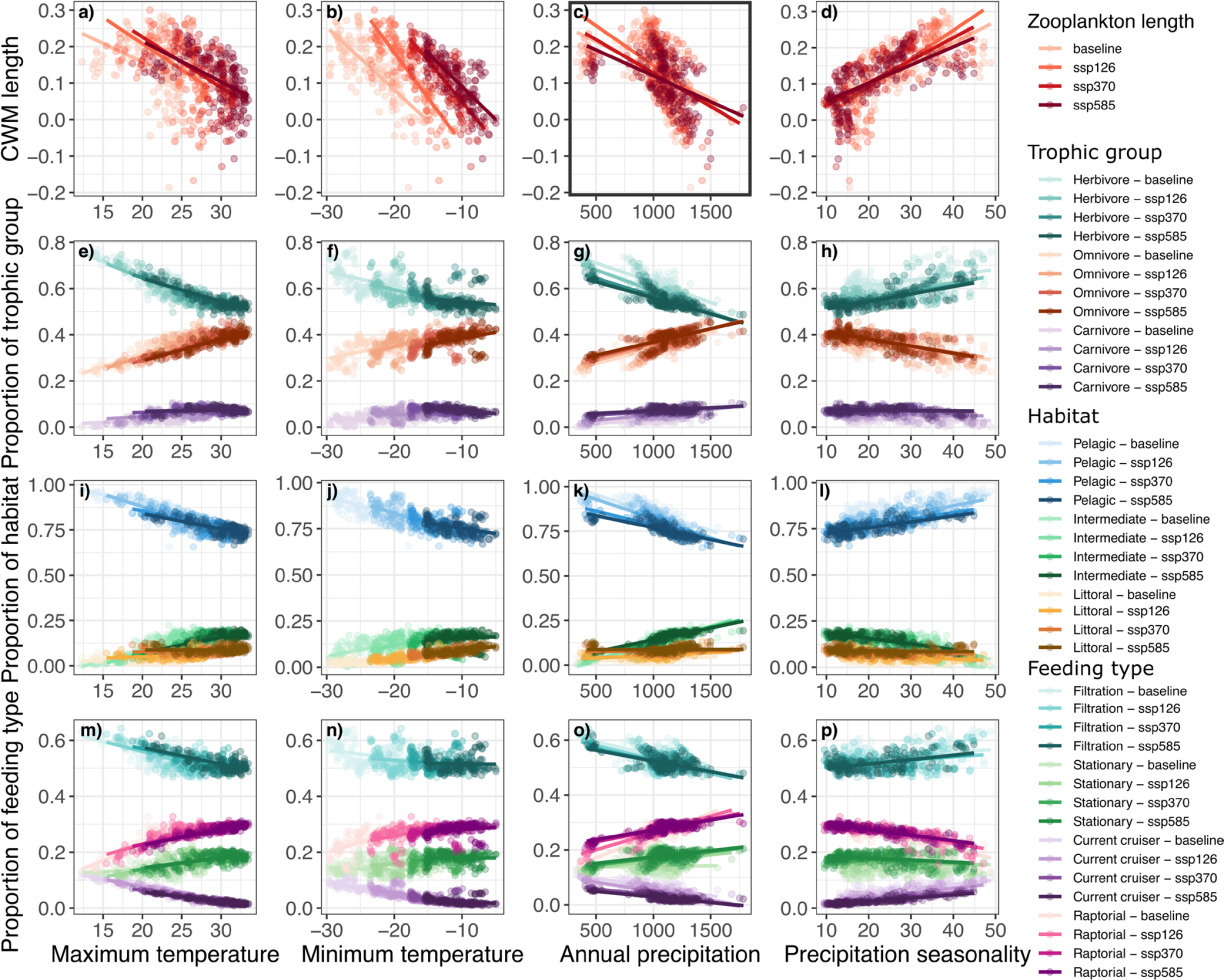
Responses of zooplankton traits to climate covariates based on the proportion of trait types (categorical traits) and community‐weighted means (CWM; quantitative traits) under baseline and future climate scenarios (SSP1–2.6, SSP3–7.0, and SSP5–8.5). Darker shades represent more extreme climate scenarios, while lighter shades represent baseline conditions. The panel with a thicker outline (c) indicates significant trait–environment relationships in baseline conditions (γ plot).

Changes in zooplankton traits along climate gradients were generally weaker than those observed for fish (Figure 7). Zooplankton body length tended to decrease slightly under warmer temperatures. Regarding the proportion of zooplankton trophic group, habitat and feeding type, trait–climate relationships remained consistent across baseline and future climate scenarios, with nearly unchanged slope. While zooplankton length was negatively associated with annual precipitation under baseline conditions, we only observed a small shift in relationship under more intense climate scenarios. However, the range of trait values narrowed in response to reduced temperature gradient lengths.

## 4 Discussion

This study provides one of the first multi‐trophic assessments of how climate change might reorganize freshwater communities, showing differential responses of fish and zooplankton to future climate scenarios. Our results show that fish and zooplankton had distinct baseline diversity patterns across the study region, although the community composition of both groups was mainly structured by climatic variables. Fish species occurrences and traits responded strongly to climate gradients, with forecasted shifts in functional traits under future climate scenarios, suggesting that fish may be more responsive to future climate change than zooplankton. Moreover, our models predict a homogenization of fish‐zooplankton co‐occurrence networks by 2100, characterized by increased connectance and reduced modularity when considering only the current pool of species. Together, these results suggest that climate change could drive substantial functional and network‐level reorganization in freshwater ecosystems at the regional scale, with potential consequences for trophic interactions and ecosystem stability.

### 4.1 Distinct Diversity and Climate Responses in Fish and Zooplankton Communities

For decades, macroecologists have sought to understand how species are distributed across landscapes and whether their occurrences overlap along environmental gradients (Gaston 2000; Leibold et al. 2004; Heino 2011). Our first objective was thus to determine whether fish and zooplankton had associated spatial *α*‐ and *β*‐diversity patterns, expecting both groups to follow energy‐ or productivity‐driven latitudinal gradients (Hillebrand 2004; Hessen et al. 2006; Pinel‐Alloul et al. 2013; Miller and Román‐Palacios 2021). Fish largely conformed to this prediction with lower richness but higher uniqueness in northern Québec. On the other hand, zooplankton deviated substantially, showing both low richness and low uniqueness in northern Québec (Figure 1). As a result, fish and zooplankton richness and uniqueness were poorly correlated across the province, with only a handful of lakes hosting significantly unique species from both groups (Figure 2). This decoupling suggests that distinct ecological processes govern the diversity of these two trophic levels, including differences in environmental filtering and habitat requirements, similar to the contrasting drivers observed among trophic groups in other systems (Liu et al. 2020). Such decoupling is not surprising given the differences in dispersal ability (high for zooplankton relative to fish), as well as generation times and population growth rates of zooplankton (mostly weeks to months) compared to fish (most annually or longer).

The decoupled pattern in fish‐zooplankton diversity was partly reflected in community composition. Although occurrences of both groups were constrained by the same environmental variables (Figure S1), the importance of these drivers differed between fish and zooplankton (Figure S2) as observed by Taranu et al. (2021) for 49 lakes in southern Québec. Overall, climatic variables (i.e., air temperature and precipitation variables) had an overarching influence, as predicted considering the relatively large spatial scale of our study (Figure 3), although fish consistently responded more strongly than zooplankton to these gradients.

Our finding that zooplankton composition is more strongly shaped by climatic gradients than its diversity aligns with previous work showing that while lake morphometry drives pan‐Canadian zooplankton diversity, water quality (including water temperature) best predicts community composition (Paquette et al. 2022). Nevertheless, fish niches, and their modulation by functional traits, were more strongly correlated with environmental variables than those of zooplankton (Figure 3, Figure S3). This result may appear counterintuitive, as the stronger dispersal limitation of fish should reduce the influence of environmental filtering relative to that observed for zooplankton (Leibold et al. 2004; Beisner et al. 2006; Taranu et al. 2021). However, our reliance on presence–absence data may have obscured stronger zooplankton–environment relationships. Indeed, zooplankton are nearly ubiquitous due to efficient dispersal and rapid colonization, yet their abundances (rather than occurrences) respond most sensitively to environmental gradients (Beisner et al. 2006; Jeppesen et al. 2011). Moreover, while many fish species have strict thermal requirements (Miller and Román‐Palacios 2021; van Denderen et al. 2023), zooplankton can persist at low densities in suboptimal environments or survive as resting eggs, buffering their occurrence from immediate environmental filtering (Hairston 1996).

### 4.2 Baseline Co‐Occurrence Patterns

In HMSC, significant residual variation represents fish‐zooplankton associations that remain statistically supported after accounting for shared environmental responses, indicating that these taxa co‐occur more or less frequently than expected by chance given their environmental niches. Such associations may reflect true biotic interactions such as predation, or shared responses to unmeasured variables. As we had hypothesized, we found several fish‐zooplankton interactions in our model, highlighting tight coupling between both taxonomic groups. Unsurprisingly, most statistically supported residual co‐responses involved cladocerans, a group typically experiencing higher fish predation pressure given its larger size and slower movements (Brooks and Dodson 1965; Mills et al. 1987), while most fish species that were involved in the significant co‐responses were classified as primary or secondary consumers (Figure 4). We also identified a few top predator fish species with significant residual associations with zooplankton. Such patterns are expected under bottom‐up processes, as juveniles of most fish species feed on zooplankton (Lomartire et al. 2021). They could also reflect trophic cascades, where large piscivorous fish indirectly benefit from smaller planktivorous fish feeding on zooplankton (Drenner and Hambright 2002). Interestingly, brook trout (SAFO) exhibited patterns opposite to those of all other fish species, likely reflecting the high prevalence of monospecific brook trout populations in our dataset. In sympatric lakes (i.e., where other fish species are present) brook trout generally adopt a more planktivorous diet than in allopatric systems, emphasizing the species' trophic niche plasticity (Bourke et al. 1997).

Whether these residual associations reflect selective feeding by fish on cladocerans (top‐down control), the role of large zooplankton in supporting planktivorous fish occurrence (bottom‐up control), or simply parallel responses to unmeasured drivers remains uncertain. Nonetheless, most residual associations were negative, which may indicate predation effects (Smith and Kirkwood 2025). Regardless of the underlying mechanism, the presence of these associations highlights the tight coupling of fish and zooplankton communities independent of the measured environmental variables. However, it is important to acknowledge that co‐occurrence patterns do not necessarily reflect direct ecological interactions, and additional studies are required to determine the exact mechanisms driving the observed associations (Blanchet et al. 2020).

### 4.3 Future Climate Driven Pattern of Community Network Homogenization

We predicted that under stronger climate forcing, future fish–zooplankton ecological networks based on predicted species occurrences would become less connected and more compartmentalized, following patterns reported for freshwater communities under climate and land use change (Pham et al. 2025). Contrary to these expectations, our results revealed signatures of biotic homogenization characterized by increased connectance and reduced modularity. This pattern appears to be primarily driven by temperature–occurrence relationships in fish, as northern lake communities increasingly resemble southern ones under climate forcing, consistent with northward range expansions of warm‐adapted species.

When comparing baseline conditions to future climate projections, networks showed an increase in significant species interactions (edges) and overall connectance by 2100, with slightly larger values under the intermediate forcing scenario SSP3.7‐0 (Figure 5). This suggests that fish–zooplankton interactions may become more widespread with warming, potentially reflecting stronger environmental filtering or shared tolerance traits driving synchronized responses (Freilich et al. 2018). Despite a stable number of significant species associations (constant number of nodes) across predicted networks, species pairs were more often gained than lost (Figure S5), indicating network rewiring and the emergence of new associations as warm‐water species start occurring in previously colder northern regions. Such rewiring can influence network specialization (Zhang et al. 2023) and is expected to influence food web structure, stability, and resilience in freshwater systems. Similar patterns have been documented in marine plankton undergoing environmental change, suggesting that climate‐driven reorganization of species associations may represent a common response across aquatic ecosystems (D'Alelio et al. 2019).

Average path length declined under future scenarios, again most under SSP3‐7.0, indicating that species became more directly connected. These shorter path lengths can suggest that networks may become more vulnerable to stressors, as disturbances can propagate more rapidly through highly connected networks (Montoya et al. 2006). Similarly, decreases in the number of community types and modularity along climate gradients point to reduced compartmentalization, and fewer tightly knit ecological sub‐networks. Such patterns typically reflect a loss of specialization and a shift toward generalist strategies (i.e., species with more associations; Wang et al. 2019). While low modularity has often been linked to greater ecosystem stability (Landi et al. 2018), other studies suggest it can reduce resilience to perturbations (Sun et al. 2025). For instance, Takemoto and Kajihara (2016) observed reduced modularity in pollination networks under climate change, suggesting this may represent an adaptive but potentially fragile response to stress.

Interestingly, the most pronounced network restructuring occurred under the intermediate scenario SSP3‐7.0 for most network parameters, although values were very similar with SSP5‐8.5. This likely reflects the non‐linear nature of species responses to environmental gradients (Anderson et al. 2022) and is broadly consistent with the intermediate disturbance hypothesis (Connell 1978). At moderate warming, many species might be displaced from their thermal optima, driving interaction rewiring, whereas under extreme forcing (SSP5‐8.5), species associations may already be saturated, resulting in slightly more stable but homogenized networks. Our findings suggest that climate forcing promotes more uniform but less modular fish–zooplankton communities as many fish species may expand their range, with the greatest network reorganization occurring under moderate climate change. These shifts in species associations under climate change could reduce the structural complexity of freshwater food webs, with possible consequences for ecosystem resilience and function (Gilbert 2009; Bonnaffé et al. 2024). It is important to note that our model assumes unlimited species dispersal (i.e., no movement barriers) and a fixed species pool (i.e., no new species introductions from southern regions). Both of these assumptions likely accentuate the predicted homogenization pattern, whereas realized future networks may include more species (nodes) and exhibit more stable modularity.

### 4.4 Changes in Functional Traits Under Climate Change

We hypothesized that warmer temperatures would favor smaller‐bodied species, as supported by global temperature‐size analyses (Daufresne et al. 2009). CWM analyses indeed followed this prediction and demonstrated a negative relationship between body length and maximum air temperature for both trophic levels. Under future climate scenarios, our projections indicate a further decline in fish mean body size within a lake, whereas zooplankton exhibited little change in their climate–size relationship. This pattern aligns with Daufresne et al. (2009), who suggested that higher trophic levels and larger species may be more sensitive to climate warming.

For most fish traits analyzed, we observed weaker trait–climate relationships and muted responses to environmental gradients under more intense climate scenarios, with reduced trait‐environment slopes and narrowed trait ranges by 2100 (Figure 5). Interestingly, while fish body‐size CWM declined with increasing temperature and along stronger predicted climate forcing, larger‐bodied fish species also tended to respond more positively than small fish species to maximum air temperature (positive γ slope). This may indicate that warm lakes support a greater number of small‐bodied species whose weaker positive responses nonetheless reduce the mean body size when averaged across the community. In other words, the observed decline in average fish body size likely reflects compositional shifts toward smaller species, in line with Bergmann's rule stating that warm regions are inhabited by a larger proportion of small size species (Bergmann 1847).

As expected, we also found an overall tendency for fish communities to become more thermally tolerant (increased CTmax) under more intense climate scenarios, suggesting a potential filtering toward more heat‐tolerant species, mirroring global patterns of thermophilization in riverine fish communities in response to climate and land use changes (Comte et al. 2021). We also observed trophic and habitat‐related shifts, including a decrease in secondary consumers and an increase in primary consumers, as well as a decline in surface feeders toward more generalist species. These patterns are consistent with our network results, as higher connectance is often linked to increased generalism (González et al. 2010), although metaweb data including prey macroinvertebrate communities would be necessary to further support this finding. Moreover, rising water temperatures have been shown to favor omnivorous fish species, potentially intensifying predation pressure on zooplankton (Jeppesen et al. 2010).

In contrast, zooplankton displayed limited changes in trait–climate relationships under future scenarios compared to baseline conditions, which is unsurprising given their weaker relationship with climate variables under baseline conditions. This stability may reflect their short generation times, high phenotypic plasticity, and the buffering effects of resting stages (Yampolsky et al. 2014; Brun et al. 2016; Santos and Ebert 2023), or it could indicate stronger sensitivity to other unmeasured stressors than to climatic ones.

Overall, fish traits appeared more responsive to climate change than did zooplankton, raising concerns of food‐web decoupling in the future. Such decoupling can result in reduced fish recruitment, altered energy transfer, and ecosystem destabilization if prey is unavailable when fish need them most (He et al. 2018; Heneghan et al. 2023). However, our models do not account for indirect effects that changing fish traits could have on zooplankton. For example, an increase in small‐bodied, planktivorous fish species as our model predicts is well‐documented to impose stronger top‐down control on zooplankton, altering community composition, reducing overall zooplankton biomass, and shifting size structure toward smaller species (e.g., Nicolle et al. 2011; Ersoy et al. 2019; Guo et al. 2023). Indeed, Gyllström et al. (2005) and Gauthier et al. (2014) demonstrated that zooplankton responses to climate forcing were largely mediated by fish predation, underscoring the importance of considering multi‐trophic interactions when assessing ecological responses to climate change.

### 4.5 Limitations, Future Directions, and Conclusions

We acknowledge several limitations to this study, including the inherent uncertainty of climate projections. Most importantly, our model assumes that functional traits are fixed within species, thereby underestimating the potential influence of climate change on intraspecific trait variability. However, it has been demonstrated that warming can drive shifts toward smaller‐bodied individuals and alter fish diets through behavioral change (Daufresne et al. 2009; Gauzens et al. 2024). Furthermore, our model assumes unlimited dispersal across Québec and a fixed species pool, excluding the potential for future colonization by southern species. We were also limited in functional trait availability particularly for zooplankton thermal tolerances, which may underestimate climate effects on their trait‐mediated responses. The importance of declining dissolved oxygen concentration under warmer water temperatures (Adrian et al. 2009; Razlutskij et al. 2018), and its influence on species niches and their traits was also overlooked in this study. Moreover, our research does not include phytoplankton communities, another key component of aquatic food webs. Previous joint species distribution models including both phytoplankton and zooplankton revealed co‐occurrence patterns driven by both environmental and biotic variables (Shi et al. 2025), and that zooplankton served as an important trophic link between phytoplankton and fish (Taranu et al. 2021). Similarly, future land use changes, such as expected increases in agriculture and urbanization in northern Québec, were not considered, though these may interact with climate drivers to further shape communities. Finally, temporal variability within biotic communities was not analyzed in this study, although it was demonstrated to have an important influence on directionally and strength of plankton–environment relationships (Bolduc et al. 2026). Future work could strengthen these analyses by incorporating phylogenetic information into HMSC, which may improve predictions of species–environment relationships (Ovaskainen et al. 2017). Expanding this framework to other ecoregions would also be valuable, since temperate lakes may not represent global patterns, and processes may vary across biogeographic contexts.

Nonetheless, these findings highlight the value of trait‐based and network analyses for predicting community responses to climate change and emphasize the contrasting sensitivities of different trophic levels in freshwater ecosystems. The HMSC framework provided a powerful way to disentangle how environmental and biotic factors jointly shape fish and zooplankton communities and how functional traits mediate their responses to climate gradients. Our results identified a trend of community homogenization under future climatic scenarios, with potential community and ecosystem level implications, including potential fish‐zooplankton decoupling as the sensitivities of both trophic levels to climate change differ. Moreover, changes in regional fish–zooplankton distribution and co‐occurrence, trait composition at the lake level, and network structure likely reflect underlying trophic reorganizations that could translate into altered ecosystem functioning at the lake scale. Ultimately, our results demonstrate that climate change impacts on freshwater food webs are complex, context‐dependent, and unlikely to conform to a “one size fits all” pattern.

## Author Contributions

**Cindy Paquette:** writing – original draft, visualization, validation, funding acquisition, formal analysis, data curation, conceptualization, methodology. **Dario J. Di Girolamo:** writing – review and editing, visualization, methodology, formal analysis. **Jennifer Pham:** writing – review and editing, visualization, formal analysis, methodology. **Otso Ovaskainen:** writing – review and editing, methodology, formal analysis, visualization. **Stéphanie Gagné:** validation, writing – review and editing, data curation. **Véronique Leclerc:** funding acquisition, supervision, writing – review and editing, conceptualization. **Beatrix E. Beisner:** conceptualization, funding acquisition, data curation, supervision, writing – review and editing, project administration. **Vincent Fugère:** conceptualization, funding acquisition, data curation, supervision, writing – review and editing, methodology, project administration. **Zofia E. Taranu:** conceptualization, investigation, funding acquisition, writing – review and editing, project administration, supervision.

## Funding

This work was supported by the Natural Sciences and Engineering Research Council of Canada (ALLRP, 582937‐23); Department of Fisheries and Oceans Canada; Groupe de Recherche Interuniversitaire en Limnologie; Ministère de l’Environnement, de la Lutte Contre les Changements Climatiques, de la Faune et des Parcs du Québec; Research Council of Finland (336212, 345110); QUADRAT DTP; and Environment and Climate Change Canada.

## Conflicts of Interest

The authors declare no conflicts of interest.

## Supporting information


**Figure S1:** Global error‐weighted conditional permutation importance across fish and zooplankton species.
**Figure S2:** Heatplot of species responses (beta parameters) to environmental predictors from the HMSC model, with red indicating positive and blue indicating negative relationships. Only statistically supported associations (posterior probability at least 0.90) are shown. Rows correspond to species, with fish species (identified by four‐letter taxonomic codes) listed first and zooplankton shown last. Full fish species names corresponding to taxonomic codes are provided in Table S1.
**Figure S3:** Heatplot of trait responses (gamma parameters) to environmental predictors from the HMSC model, displaying standardized regression coefficients, with red indicating positive and blue indicating negative relationships. Only statistically supported associations (posterior probability at least 0.90) are shown.
**Figure S4:** Chord Diagram showing positive (a; red) and negative (b; blue) HMSC significant residual associations between fish (orange) and zooplankton (blue).
**Figure S5:** Barplot of the number of changes in species co‐occurrences (edges) in future climate scenarios (SSP1‐2.6, SSP3‐7.0, SSP5‐8.5) compared to baseline conditions.
**Figure S6:** Observed fish richness along baseline maximum air temperature gradient. Color saturation represents mean fish length for each lake.
**Table S1:** Fish code names with associated family, genus, species and common name.

## Data Availability

The data that support the findings of this study are available in Zenodo at 10.5281/zenodo.17916221 (Paquette, Gagné, and Fugère 2025).
