# Supplementary material for: Fish and Zooplankton Co‐Responses to Environmental Gradients Under Different Climate Change Scenarios
Source: Glob Chang Biol. 2026 Apr 16;32(4):e70845. doi: 10.1111/gcb.70845 (PMC13087492; doi:10.1111/gcb.70845)
Supplement: Supplementary file 1 — Figure S1: Global error‐weighted conditional permutation importance across fish and zooplankton species. Figure S2: Heatplot of species responses (beta parameters) to environmental predictors from the HMSC model, with red indicating positive and blue indicating negative relationships. Only statistically supported associations (posterior probability at least 0.90) are shown. Rows correspond to species, with fish species (identified by four‐letter taxonomic codes) listed first and zooplankton shown last. Full fish species names corresponding to taxonomic codes are provided in Table S1. Figure S3: Heatplot of trait responses (gamma parameters) to environmental predictors from the HMSC model, displaying standardized regression coefficients, with red indicating positive and blue indicating negative relationships. Only statistically supported associations (posterior probability at least 0.90) are shown. Figure S4: Chord Diagram showing positive (a; red) and negative (b; blue) HMSC significant residual associations between fish (orange) and zooplankton (blue). Figure S5: Barplot of the number of changes in species co‐occurrences (edges) in future climate scenarios (SSP1‐2.6, SSP3‐7.0, SSP5‐8.5) compared to baseline conditions. Figure S6: Observed fish richness along baseline maximum air temperature gradient. Color saturation represents mean fish length for each lake. Table S1: Fish code names with associated family, genus, species and common name. [file GCB-32-e70845-s001.docx]

Supplementary material

Fish and zooplankton co-responses to environmental gradients under different climate change scenarios

**Figure S1**. Global error-weighted conditional permutation importance across fish and zooplankton species

**Figure S2**. Heatplot of species responses (beta parameters) to environmental predictors from the HMSC model, with red indicating positive and blue indicating negative relationships. Only statistically supported associations (posterior probability at least 0.90) are shown. Rows correspond to species, with fish species (identified by four-letter taxonomic codes) listed first and zooplankton shown last. Full fish species names corresponding to taxonomic codes are provided in Table S1.


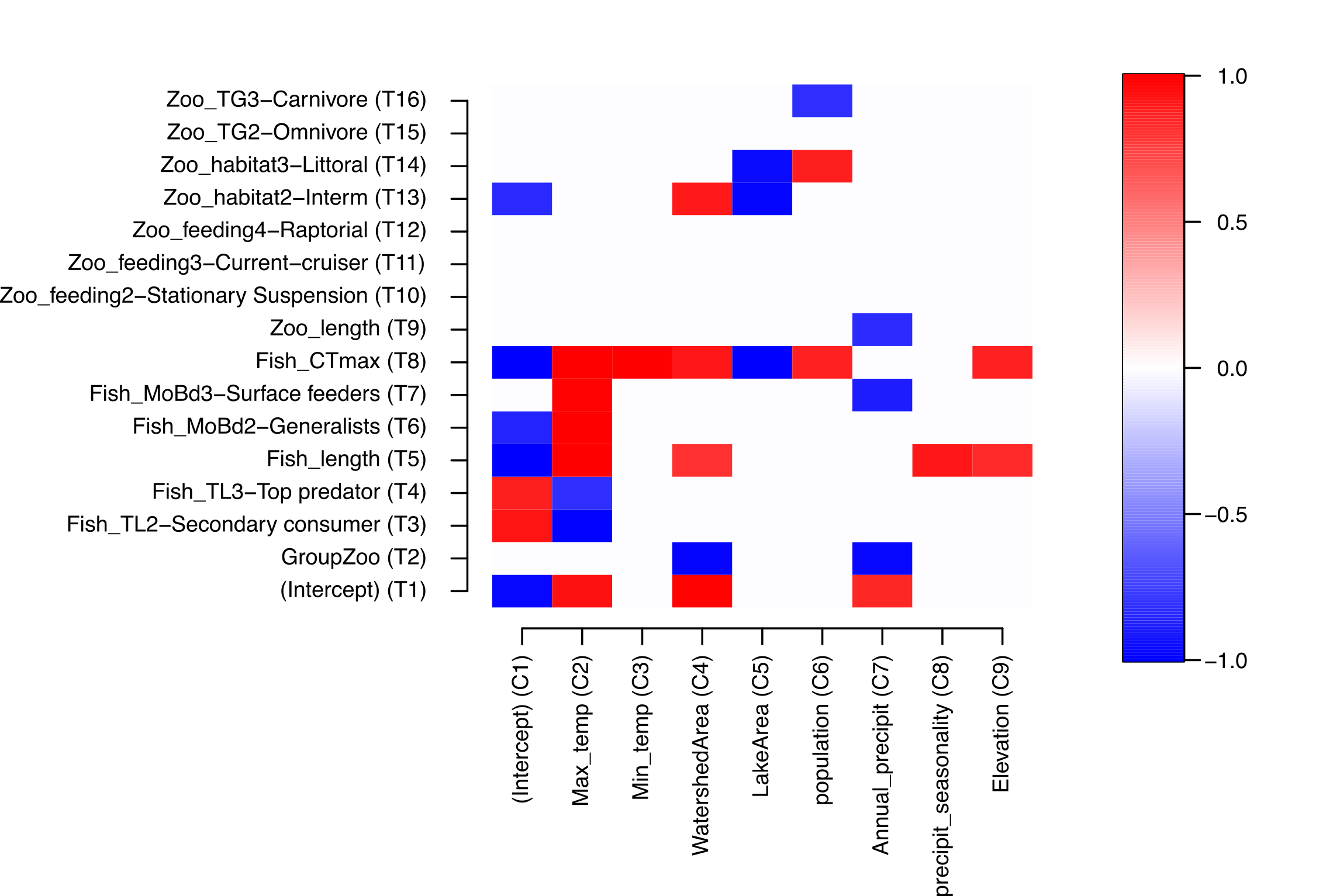


**Figure S3**. Heatplot of trait responses (gamma parameters) to environmental predictors from the HMSC model, displaying standardized regression coefficients, with red indicating positive and blue indicating negative relationships. Only statistically supported associations (posterior probability at least 0.90) are shown.

**
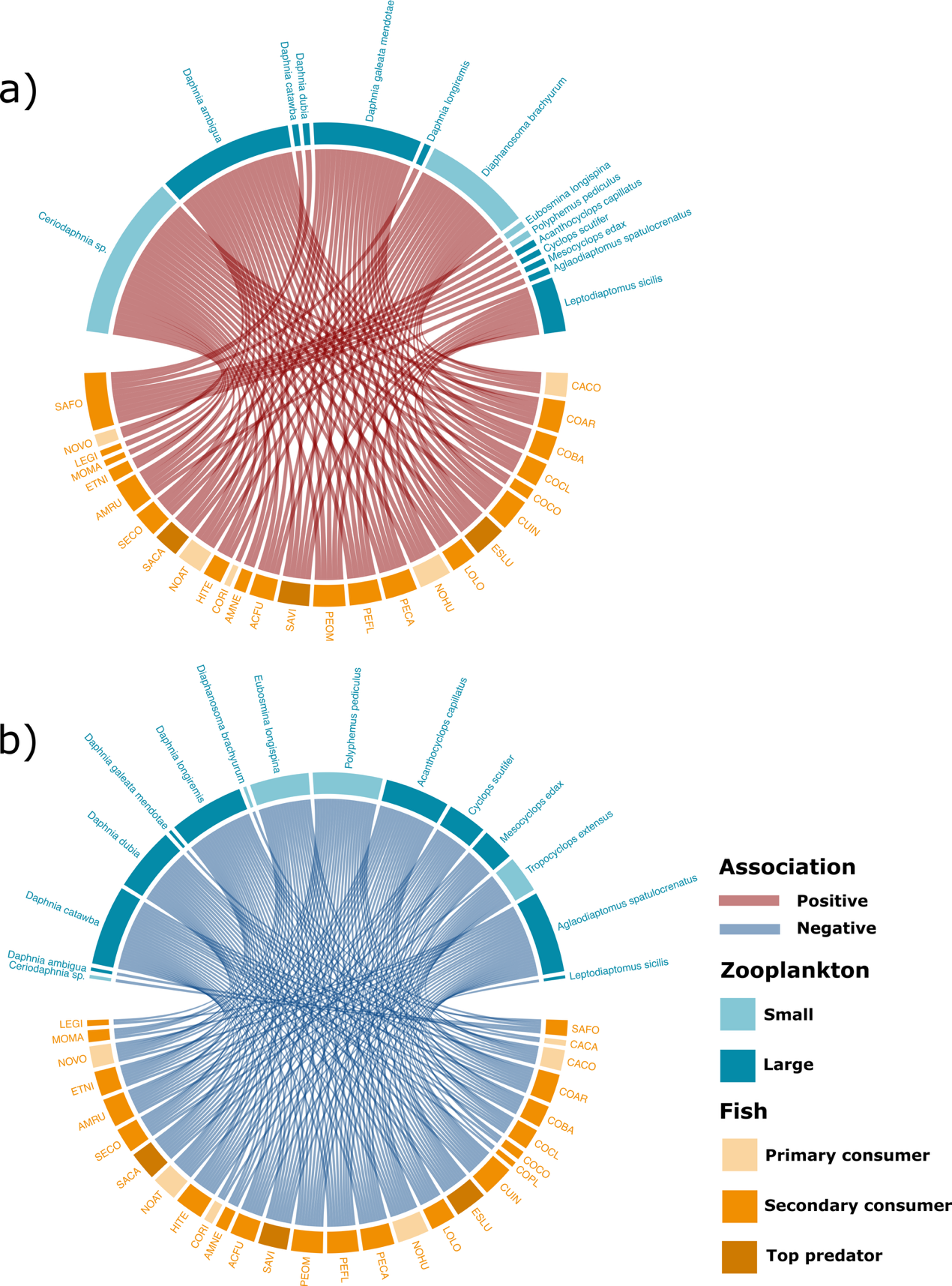
**

**Figure S4.** Chord Diagram showing positive (a; red) and negative (b; blue) HMSC significant residual associations between fish (orange) and zooplankton (blue).

**Figure S5**. Barplot of the number of changes in species co-occurrences (edges) in future climate scenarios (SSP1-2.6, SSP3-7.0, SSP5-8.5) compared to baseline conditions.

**Figure S6**. Observed fish richness along baseline maximum air temperature gradient. Color saturation represents mean fish length for each lake.

**Table S1**. Fish code names with associated family, genus, species and common name

| Code | Family | Genus | Species | Common name |
| --- | --- | --- | --- | --- |
| ACFU | Acipenseridae | Acipenser | fulvescens | lake sturgeon |
| AMNE | Ictaluridae | Ameiurus | nebulosus | Brown bullhead |
| AMRU | Centrarchidae | Ambloplites | rupestris | Rock bass |
| ANRO | Anguillidae | Anguilla | rostrata | American eel |
| CACA | Catostomidae | Catostomus | catostomus | Longnose sucker |
| CACO | Catostomidae | Catostomus | commersonii | White sucker |
| CHEO | Cyprinidae | Chrosomus | eos | Northern redbelly dace |
| CHNE | Cyprinidae | Chrosomus | neogaeus | Finescale dace |
| COAR | Salmonidae | Coregonus | artedi | Cisco |
| COBA | Cottidae | Cottus | bairdii | mottled sculpin |
| COCL | Salmonidae | Coregonus | clupeaformis | Lake whitefish |
| COCO | Cottidae | Cottus | cognatus | slimy sculpin |
| COPL | Cyprinidae | Couesius | plumbeus | Lake chub |
| CORI | Cottidae | Cottus | ricei | Spoonhead sculpin |
| CUIN | Gasterosteidae | Culaea | inconstans | brook stickleback |
| CYCA | Cyprinidae | Cyprinus | carpio | common carp |
| CYSI | Cyprinidae | Cyprinella | spiloptera | spotfin shiner |
| ESMA | Esocidae | Esox | masquinongy | muskellunge |
| ESLU | Esocidae | Esox | lucius | Northern pike |
| ESNI | Esocidae | Esox | niger | chain pickerel |
| ETNI | Percidae | Etheostoma | nigrum | Johnny darter |
| ETOL | Percidae | Etheostoma | olmstedi | tessellated darter |
| FUDI | Fundulidae | Fundulus | diaphanus | banded killifish |
| GAAC | Gasterosteidae | Gasterosteus | aculeatus | threespine stickleback |
| HIAL | Hiodontidae | Hiodon | alosoides | goldeye |
| HITE | Hiodontidae | Hiodon | tergisus | Mooneye |
| HYRE | Cyprinidae | Hybognathus | regius | Eastern silvery minnow |
| LEGI | Centrarchidae | Lepomis | gibbosus | Pumpkinseed |
| LOLO | Lotidae | Lota | lota | Burbot |
| LUCO | Cyprinidae | Luxilus | cornutus | Common shiner |
| MAMA | Cyprinidae | Margariscus | margarita | Pearl dace |
| MIDO | Centrarchidae | Micropterus | dolomieu | Smallmouth bass |
| MISA | Centrarchidae | Micropterus | salmoides | largemouth bass |
| MOAM | Moronidae | Morone | americana | white perch |
| MOAN | Catostomidae | Moxostoma | anisurum | silver redhorse |
| MOMA | Catostomidae | Moxostoma | macrolepidotum | shorthead redhorse |
| MOVA | Catostomidae | Moxostoma | valenciennesi | greater redhorse |
| NOAT | Cyprinidae | Notropis | atherinoides | emerald shiner |
| NOBI | Cyprinidae | Notropis | bifrenatus | bridle shiner |
| NOCR | Cyprinidae | Notemigonus | crysoleucas | Golden shiner |
| NOHD | Cyprinidae | Notropis | heterodon | blackchin shiner |
| NOHL | Cyprinidae | Notropis | heterolepis | blacknose shiner |
| NOHU | Cyprinidae | Notropis | hudsonius | Spottail shiner |
| NORU | Cyprinidae | Notropis | rubellus | rosyface shiner |
| NOST | Cyprinidae | Notropis | stramineus | sand shiner |
| NOVO | Cyprinidae | Notropis | volucellus | mimic shiner |
| ONMY | Salmonidae | Oncorhynchus | mykiss | rainbow trout |
| OSMO | Osmeridae | Osmerus | mordax | Rainbow smelt |
| PECA | Percidae | Percina | caprodes | Logperch |
| PEFL | Percidae | Perca | flavescens | Yellow perch |
| PEOM | Percopsidae | Percopsis | omiscomaycus | Trout-perch |
| PHEO | Cyprinidae | Phoxinus | eos | Northern redbelly dace |
| PHNE | Cyprinidae | Phoxinus | neogaeus | finescale dace |
| PINO | Cyprinidae | Pimephales | notatus | bluntnose minnow |
| PIPR | Cyprinidae | Pimephales | promelas | Fathead minnow |
| PRCY | Salmonidae | Prosopium | cylindraceum | Round whitefish |
| PUPU | Gasterosteidae | Pungitius | pungitius | ninespine stickleback |
| RHAT | Cyprinidae | Rhinichthys | atratulus | Eastern blacknose dace |
| RHCA | Cyprinidae | Rhinichthys | cataractae | longnose dace |
| UMLI | Umbridae | Umbra | limi | central mudminnow |
| SAAL | Salmonidae | Salvelinus | alpinus oquassa | landlocked Arctic char |
| SACA | Percidae | Sander | canadensis | Sauger |
| SAFO | Salmonidae | Salvelinus | fontinalis | Brook trout |
| SANA | Salmonidae | Salvelinus | namaycush | lake trout |
| SASO | Salmonidae | Salmo | salar | landlocked salmon |
| SATR | Salmonidae | Salmo | trutta | brown trout |
| SAVI | Percidae | Sander | vitreus | Walleye |
| SEAT | Cyprinidae | Semotilus | atromaculatus | Creek chub |
| SECO | Cyprinidae | Semotilus | corporalis | Fallfish |
